# The Dopamine D3 Receptor Knockout Mouse Mimics Aging-Related Changes in Autonomic Function and Cardiac Fibrosis

**DOI:** 10.1371/journal.pone.0074116

**Published:** 2013-08-30

**Authors:** Tracy L. Johnson, David A. Tulis, Benjamin E. Keeler, Jitka A. Virag, Robert M. Lust, Stefan Clemens

**Affiliations:** Brody School of Medicine, Department of Physiology, East Carolina University, Greenville, North Carolina, United States of America; Universityhospital Düsseldorf, Germany

## Abstract

Blood pressure increases with age, and dysfunction of the dopamine D3 receptor has been implicated in the pathogenesis of hypertension. To evaluate the role of the D3 receptor in aging-related hypertension, we assessed cardiac structure and function in differently aged (2 mo, 1 yr, 2 yr) wild type (WT) and young (2 mo) D3 receptor knockout mice (D3KO). In WT, systolic and diastolic blood pressures and rate-pressure product (RPP) significantly increased with age, while heart rate significantly decreased. Blood pressure values, heart rate and RPP of young D3KO were significantly elevated over age-matched WT, but similar to those of the 2 yr old WT. Echocardiography revealed that the functional measurements of ejection fraction and fractional shortening decreased significantly with age in WT and that they were significantly smaller in D3KO compared to young WT. Despite this functional change however, cardiac morphology remained similar between the age-matched WT and D3KO. Additional morphometric analyses confirmed an aging-related increase in left ventricle (LV) and myocyte cross-sectional areas in WT, but found no difference between age-matched young WT and D3KO. In contrast, interstitial fibrosis, which increased with age in WT, was significantly elevated in the D3KO over age-matched WT, and similar to 2 yr old WT. Western analyses of myocardial homogenates revealed significantly increased levels of pro- and mature collagen type I in young D3KO. Column zymography revealed that activities of myocardial MMP-2 and MMP-9 increased with age in WTs, but in D3KO, only MMP-9 activity was significantly increased over age-matched WTs. Our data provide evidence that the dopamine D3 receptor has a critical role in the emergence of aging-related cardiac fibrosis, remodeling, and dysfunction.

## Introduction

Normal aging is a complex composite of several different processes that can be distinguished from age-related diseases, and it is associated in humans with a gradually increasing risk of cardiovascular disease [1], [2] including hypertension [3], [4]. While the cardiovascular autonomic changes related to aging are well described both functionally [5] and morphologically [6], emerging data suggest that cardiac aging is also associated with ventricular hypertrophy and fibrosis [7], caused by reduced matrix proteoloysis and progressive deposition of collagen in the interstitial space [7], [8].

A role for the neuromodulator dopamine (DA) in the pathophysiology of hypertension has been well established [9], [10]. DA acts through two different receptor subfamilies, D1-like (D1, D5) and D2-like (D2, D3, D4), and DA usually has lower affinity for D1-like versus D2-like receptors. There is evidence for a role of both D1 [11], [12] and D3 receptor-mediated effects in the pathophysiology of hypertension [13], [14], [15], [16]. The D3 receptor is expressed in the heart [17], and several mechanisms have been reported by which failure of the D3 signaling system in the periphery might account for an increase in blood pressure [16], [18], [19]. These include interaction with the D1 receptor subtype [20], angiotensin II [21], endothelin binding receptors [22], or the G protein-coupled receptor kinase 4 in the kidney or Na^+^ transporters in the renal tube [23], [24].

Based on the established association between aging and the development of hypertension and considering observations that the D3 receptor knockout mouse (D3KO) expresses a hypertensive phenotype [16], we hypothesized that a dysfunction of the D3 receptor signaling pathway is associated with the cardiac fibrosis and dysfunction observed with normal aging and that it can mimic age-dependent changes of the cardiovascular system in these animals. Using non-invasive *in vivo* blood pressure measurements, functional echocardiography, *in vitro* histology and expression and activity assays on hearts of differently-aged WT (2 mo, 1 yr, 2 yr) and young (2 mo) D3KO mice, we found that aging-related changes in blood pressure and cardiac fibrosis in WT are largely mimicked in young D3KOs. Intriguingly, these blood pressure and echocardiography data resemble aging-related changes observed in healthy, senescent humans. We propose that in aged WT and young D3KO mice, these changes occur through a collagen type I/matrix metalloproteinase-9 (MMP-9)-mediated mechanism. Based on these novel findings, we introduce the idea that the dopamine D3 receptor serves as a regulator for aging-related cardiac fibrosis and dysfunction. Thus D3KOs may provide a new experimental model with which to understand the role of DA/D3 receptor signaling in the aging heart.

## Materials and Methods

### Animals and housing

All experimental procedures were approved by the East Carolina University Animal Care and Use Committee according to the National Institutes of Health Guide for the Care and Use of Laboratory Animals (US NIH, Pub. No. 85-23, rev. 1996), and all efforts were made to minimize animal suffering. Experimental animals were divided into three age groups: wild type (WT) mice (C57BL/6): 2 mo (n = 10), 1 yr (n = 12), and 2 yr (n = 10). D3KO mice on a C57BL/6 background (B6.129S4-*Drd3^tm1Dac^*/J, n = 10) were obtained from Jackson Laboratory, Bar Harbor, ME, and maintained as a breeding colony at ECU. Animals were kept under identical conditions in the Brody School of Medicine Animal Facility with a 12∶12 h light-dark cycle and free access to standard mouse chow and water. Two additional cohorts of animals were used to assess potential differences in mortality between the two strains (WT: n = 14; D3KO: n = 15).

### Blood pressure measurements

Systolic and diastolic blood pressures and heart rates were measured in conscious mice using a non-invasive tail cuff measurement system (Model SC1000 Blood Pressure Analysis System, Hatteras Instruments, Cary, NC). Animals were placed on a warming platform (37–38°C), and a magnetic metal holder was used to restrict movement. A tail-cuff occluder was placed proximally on the tail, which was then immobilized with tape in a V-shaped block between a light source above and a photoresistor below with a LED assembly to detect the pulse. Data were displayed, recorded (SC1000 Comm Windows, version 2.50) and exported to SigmaPlot (v. 11; Systat Software, Inc., Chicago, IL) for analysis. The minimum pulse amplitude was set to 20%, the systolic threshold to 5%, and the maximum pressure to occlude blood flow in the tail to 200 mmHg.

Recording sessions usually lasted less than 10 minutes to complete per animal and consisted of 5 acclimatization cycles and 10 measurement cycles for systolic and diastolic blood pressure and heart rate. Measurements were performed at similar times of day to maintain consistency and to avoid circadian perturbations. Only animals of the same age group or strain were measured on a given day, and recording sessions were repeated daily, typically for 4–5 days.

### Functional echocardiography

Animals underwent high-resolution echocardiography (Vevo 2100, VisualSonics, Toronto, ON) using a 30 MHz transducer. All behavioral experiments were performed at room temperature. Following protocols described elsewhere [25], animals were anesthetized (2% isoflurane) and placed on a platform under a fume hood where the anesthesia was maintained via a nose cone. The chests were depilated (Nair®) to minimize ultrasound attenuation, and a contact gel (Aquasonic, Parker Laboratories, Fairfield, NJ) was applied to the thorax surface to optimize visibility of the heart. Parasternal long- and short-axis views were acquired in M-mode, and the system software then measured or calculated off-line the following parameters: systolic and diastolic diameters, ejection fraction, fractional shortening, left ventricle (LV) mass, LV mass/body weight, systolic and diastolic volume, stroke volume, cardiac output, and heart rate. While the functional echocardiography cannot measure cardiac chamber pressures and volumes, this non-invasive approach permitted us to obtain repeated datasets from the animals without fear of jeopardizing the measurements of these baseline parameters by additional cardiac catheterization.

### Tissue harvesting and histology

After *in vivo* data were acquired, animals were deeply anesthetized by exposure to isoflurane and decapitated. Hearts were quickly harvested and either snap-frozen in liquid nitrogen (n = 5 per group and strain, LV only) for protein analyses or fixed in zinc-based fixative (n = 5 per group and strain, whole heart), processed, and embedded according to routine procedures for histological analyses [26].

For morphometric analyses of left ventricle (LV) area, chamber area, and myocyte cross-sectional area (MCSA), slides were stained with hematoxylin and eosin. Briefly, for ventricular and chamber areas, 4 sections per heart were imaged at 20×, the measurements were averaged per animal, and each of those averages was used to calculate the mean and variation for all hearts in each group. For MCSA, 3 images in both the epicardial and endocardial regions of 2 sections per hearts were taken. In each image, the area of 3–8 myocytes with centrally located nuclei was measured and recorded. The average of these numbers were calculated for each animal and then those numbers were averaged for n = 5 per group [27].

### Fibrosis staining

Picrosirius Red/Fast Green staining protocol was performed according to routine procedures in order to quantify interstitial collagen content in the LV [28]. Briefly, slides were de-paraffinized and rehydrated, immersed in a 0.1% picrosirius red/fast green solution (Sigma-Aldrich, 365548, F7258, P6744) for 30 min, cleared and coverslipped [29]. Using Adobe Photoshop software (Adobe Systems, Mountain View, CA), red collagen fibril pixels were then counted and expressed as a percentage of the total number of pixels (total  =  green + red − white).

### Immunoprecipitation

Mouse WT and D3KO whole heart lysates were diluted to 1 µg/ul total protein (in PBS) and pre-cleared with protein A-agarose/sepharose beads (50%, v/v in PBS). Beads were removed and the supernatants were incubated in immunoprecipitating antibody overnight at 4°C, after which the immunocomplex was captured with protein A beads. Protein A-antigen complexes were pelleted by centrifugation at 14,000 rpm for 5 seconds. Pellets were washed 3× in ice cold PBS, the beads resuspended, boiled (5 min), centrifuged (5–7 minutes), and the immunocomplexes were resolved by SDS-PAGE followed by Western blotting for collagen types I, III, and IV.

### Western blotting

Western blotting was performed as routine for collagen types I, III, and IV [30], [31]. Briefly, prepared immunocomplexes were transferred to nitrocellulose membrane, blocked and incubated with antibodies directed against collagen types I and IV (1∶500 each, Santa Cruz) and type III (1∶1000; Abcam) overnight at 4°C. After washes the membranes were incubated in anti-rabbit horseradish peroxidase-conjugated secondary antibodies (1∶1000) for 1 hr at room temperature. Blots were washed and incubated in Pierce enhanced chemoluminescence reagents (Thermo Scientific) and exposed to photographic film. Densitometry was performed using ImageJ software (version 1.45 s, National Institutes of Health). Blots were stripped of primary antibodies at 50°C for 30 minutes using a stripping solution containing 10% SDS and 100 mM 2-mercaptoethanol in 62.5 mM Tris buffer (pH 6.8), and re-probed for α-tubulin. Data are represented as collagen expression normalized to alpha-tubulin within each sample.

### Column zymography

Column gel zymography was performed as previously described [32], [33] to assess gelatinolytic activities of MMP-2 and MMP-9. Secreted MMP activities from whole heart homogenates were assessed using Novex 10% Tris-glycine zymogram gels containing 0.1% gelatin and using XCell *SureLock* Mini-Cells (Invitrogen). Briefly, samples were denatured in SDS buffer under non-reducing conditions and then electrophoresed in the presence of SDS running buffer. Samples were renatured, equilibrated, destained, and regions of MMP activity were quantified with ImageJ.

### Animal survival

In order to determine survival of WT and D3KO mice, a Kaplan-Meier analysis was performed using terminal morbidity or mortality as the endpoint. An additional 14 male WT and 15 male D3KO mice were used for these studies.

### Statistical analysis

All data are presented as mean ± standard error of the mean (SEM). SigmaPlot 11 (Systat Software, Inc., Chicago, IL) was used to analyze data and to test for significant differences in the course of an experiment. Unless otherwise stated, a One-way Analysis of Variance (ANOVA) was performed on data at a minimum p<0.05 threshold, and appropriate post-hoc tests were applied to evaluate differences between treatment groups.

## Results

### Blood pressure & heart rate measurements

Non-invasive blood pressure and heart rate recordings were obtained from a total of 36 animals: 2 mo WT (n = 10), 1 yr WT (n = 12), 2 yr WT (n = 8), and 2 mo D3KO (n = 6). On average, animals in each group were subjected to 4–5 recording sessions, with the first 2–3 sessions used to acclimate the animals and the last 3 sessions used for data analysis.

**Figure 1:**
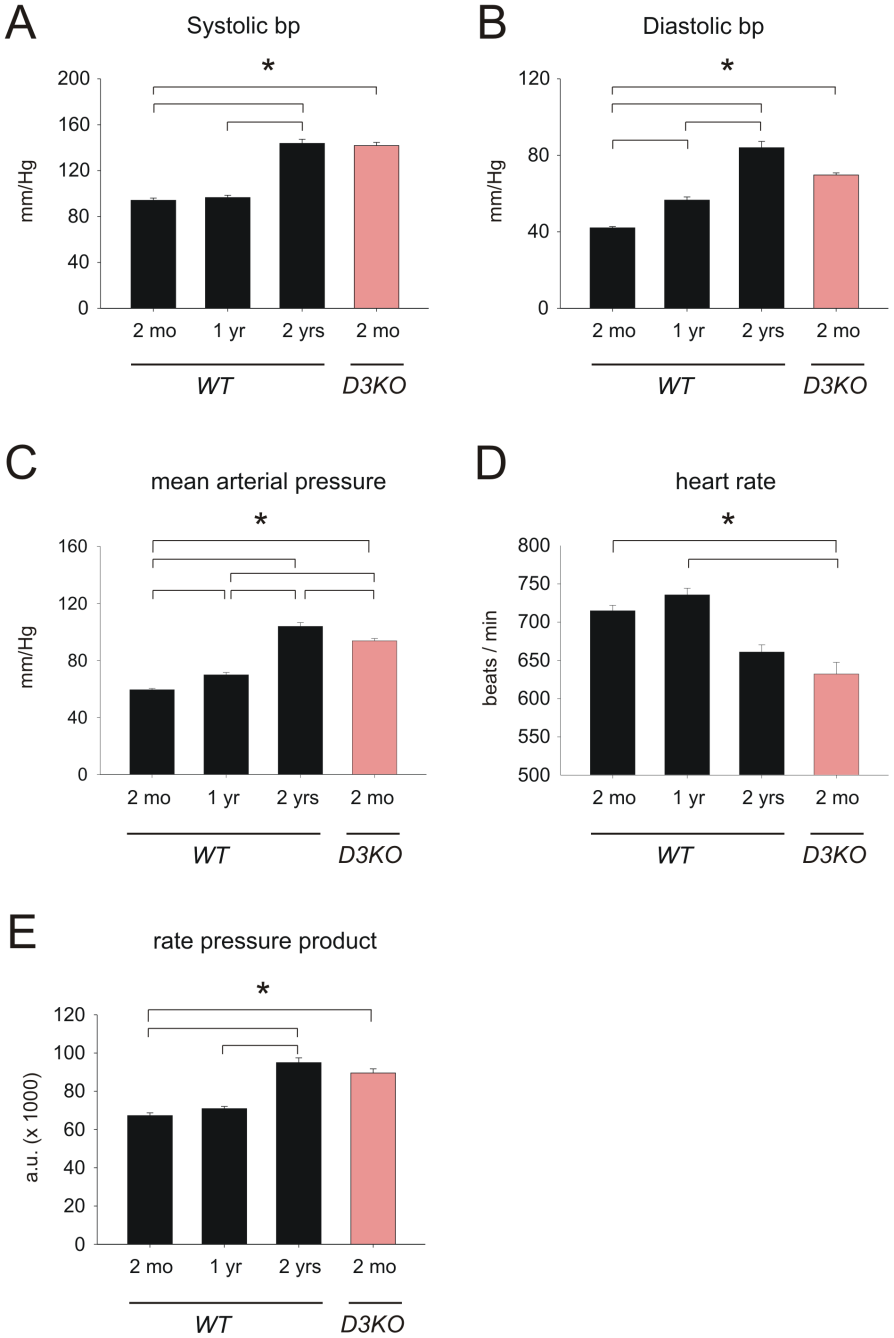
Blood pressure, heart rate, and rate-pressure product in WT and D3KO. **A.** Systolic blood pressure is stable in WT from 2 mo to 1 yr, but significantly increases from 1 yr to 2 yr (p<0.001, ANOVA). D3KO show similar values to 2 yr old WT. **B.** Diastolic blood pressure increases gradually with age in WT and reaches its peak at 2 years of age. D3KO show levels comparable to those of the 2 yr old WT and significantly higher than 2 mo or 1 yr old WT (p<0.001, ANOVA). **C.** Mean arterial pressure (MAP) increases gradually with age in WT and reaches its peak at 2 years of age. D3KO show levels comparable to those of the 2 yr old WT and significantly higher than 2 mo or 1 yr old WT (p<0.001, ANOVA). **D.** Heart rate reaches a peak in 1 yr old WT and drops significantly in the 2 yr old WT, and values are similar between 2 yr old WT and 2 mo old D3KO. **E.** The rate-pressure product increases with age in WT and is similar between 2 yr old WT and 2 mo old D3KO.

In WT animals, aging was accompanied by significant increases in systolic and diastolic blood pressures (Figs. 1A, 1B, respectively). Systolic values were similar between the 2 mo and 1 yr old WT animals (94.17±1.94 mmHg and 96.57±1.95 mmHg, respectively), but increased significantly by year 2 to 143.76±3.57 mmHg (p<0.001, Fig. 1A). In comparison, diastolic values showed a significant, age-dependent increase from 42.09±0.65 mmHg at 2 months to 56.59±1.67 at 1 year to 83.97±3.3 at 2 years of age in the WT mice (p<0.001, Fig. 1B). Intriguingly, systolic blood pressure in the young 2 mo old D3KO (141.85±2.72 mmHg) was comparable to that of the 2 yr old WT mice and was significantly higher than for the 2 mo old WT (Fig. 1A). Similarly, diastolic blood pressures of the young 2 mo old D3KO (69.71±1.1 mmHg) were comparable to those of the 2 yr old WTs and were significantly higher than the age-matched WT (Fig. 1B, p<0.001).

Increases in blood pressures with age in WT mice were paralleled by a corresponding significant increase in mean arterial pressure (MAP) from 59.4±0.9 mmHg at 2 mo over 69.9±1.7 mmHg at 1 yr, to 103.9.85±2.6 mmHg at 2 yr (Fig. 1C, p<0.001, ANOVA). MAPs of the young D3KO animals were slightly, albeit significantly, lower than those of the 2 yr old WT (93.8±1.6 mmHg), but still significantly higher than of both 2 mo and 1 yr old WTs (p<0.001, ANOVA).

Increases in blood pressures and MAP with age in the WT mice were accompanied by a strong decrease in heart rate from 714.88±6.96 beats-per-minute (bpm) at 2 mo to 660.85±9.3 bpm at 2 yr (Fig. 1D). This bradycardia in the 2 yr old WT animals was mimicked in the young 2 mo D3KOs (632±15.32 bpm, p<0.001, Fig. 1D). To assess myocardial oxygen requirements and work load, rate pressure product (RPP) was calculated as a product of systolic blood pressure x heart rate (Fig. 1E). The RPP in WT mice rose from 67302±1430 at 2 mo over 70936±1151 at 1 yr to 94974±2530 at 2 yrs (+41%; p<0.001). The RPP in 2 mo old D3KO was comparable to that of the 2 yr old WT but was significantly increased (33%; p<0.001) over age-matched WT controls (89565±2176; Fig. 1E). Together, these data provide evidence that the aging-related changes in blood pressure and heart rate affiliated with old WT are already present in young D3KO mice.

Table 1 lists body weights for the four animal cohorts in our study. In WT, we observed a significant increase in body weight with age with a peak at a 1 year (p<0.033, ANOVA); however, we did not detect differences between 2 mo old WT and age-matched D3KOs. This suggests that the observed differences in blood pressure, heart rate, RPP, and MAP between the age-matched WT and D3KO did not arise from differences associated with body weight.

**Table 1:** Animal weights.

	2 mo WT	1 yr WT	2 yrs WT	2 mo D3KO	*ANOVA*
Body weight (g)	26.83±1.48	42.49±6.98♯	35.15±3.57	28.98±1.99	p = 0.033

Average values ±S.E.

♯  =  significant difference between 2 mo WT and 1 yr WT.

### Functional echocardiography

**Figure 2:**
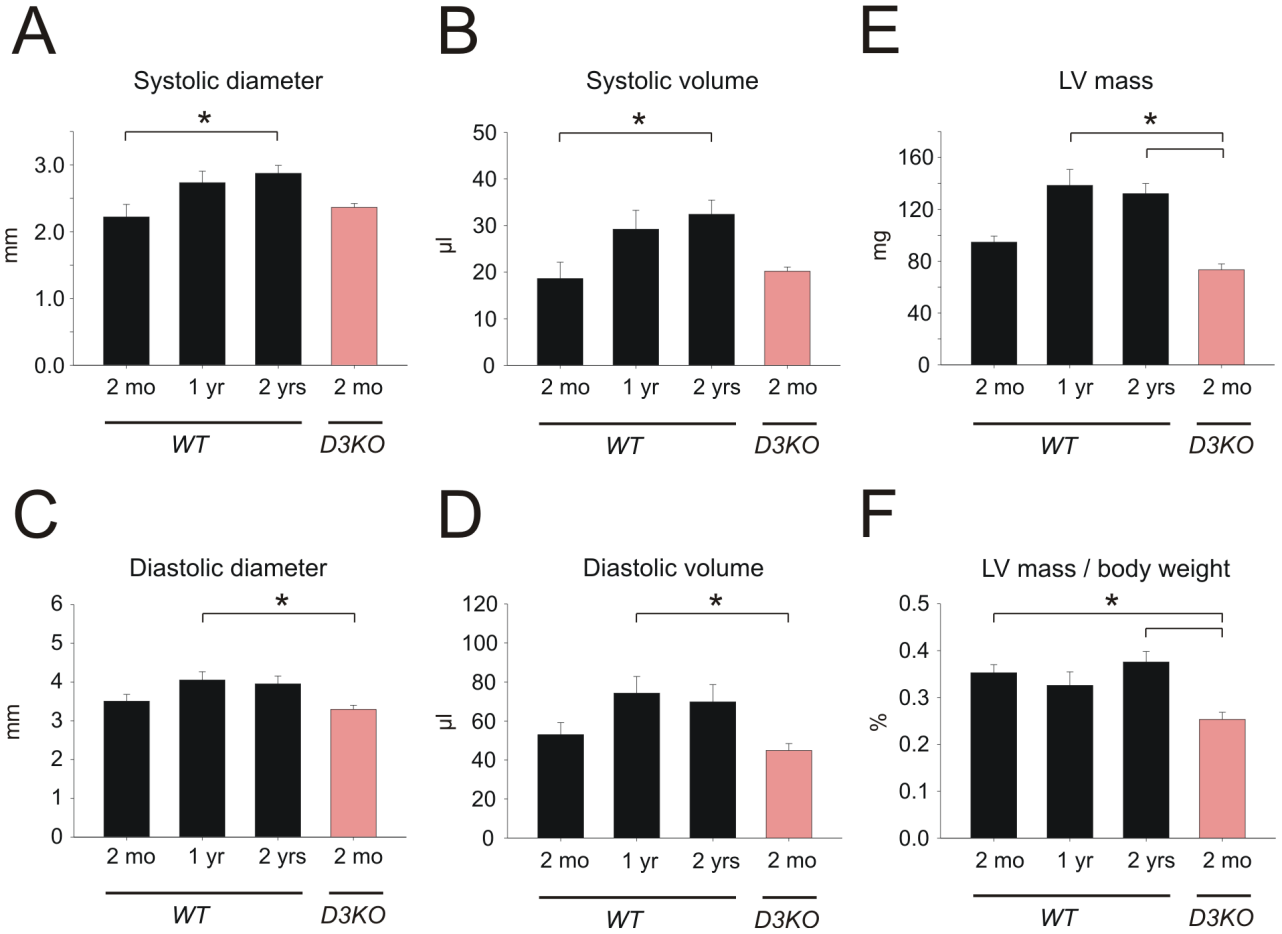
Changes in heart morphology with age. **A1.** In WT, systolic diameter increases with age and is significantly larger in 2 yr old WT compared to 2 mo. Systolic diameter in D3KO is not significantly different from age-matched WT. **A2.** In WT, diastolic diameter slightly increases with age and is significantly larger in 1 yr old WT compared to 2 mo old D3KO. Diastolic diameter in D3KO is not significantly different from age-matched WT. **B1.** In WT, systolic volume increases with age and is significantly larger in the 2 yr WT compared to the 2 mo old WT, but systolic volumes of age-matched WT and D3KO are similar. **B2.** In WT, diastolic volume increases with age and is significantly larger in 1 yr old WT over 2 mo, but diastolic volumes of young WT and D3KO are similar. **C1.** Left ventricle (LV) mass increases with age in WT and is significantly larger in 1 and 2 yr old WT compared to 2 mo old WT; however, LV masses between age-matched WT and D3KO are not significantly different. **C2.** In WT, LV mass adjusted for body weight remains stable with age but is significantly decreased in 2 mo old D3KO compared to 2 yr and 2 mo old WT.

**Figure 3:**
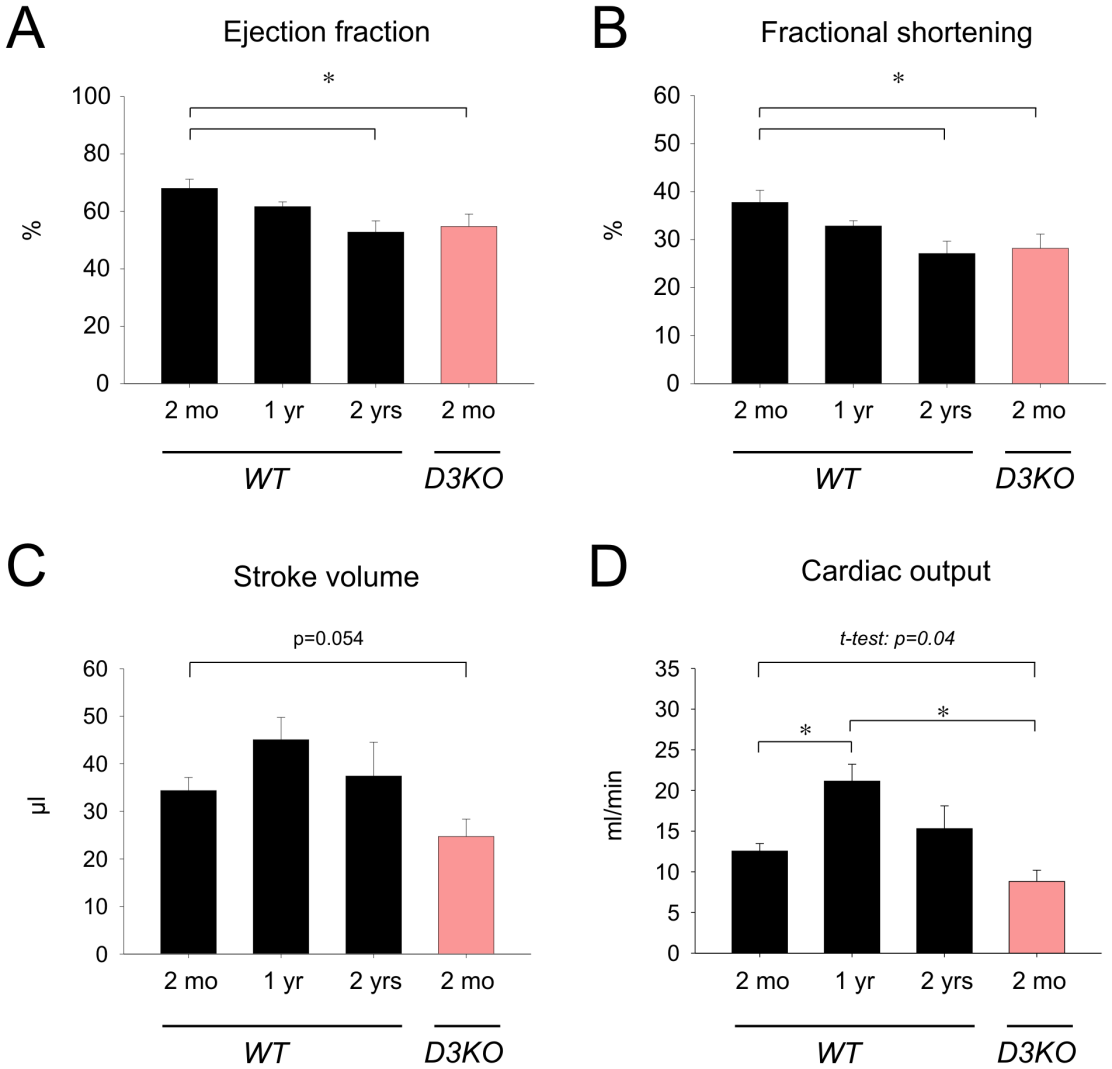
Changes in heart function with age. **A.** In WT, ejection fraction decreases with age and is significantly reduced in 2 yr old WT compared to 2 mo. Ejection fraction in D3KO is significantly reduced from age-matched WT and is comparable to that of the 2 yr old WT. **B.** In WT, fractional shortening decreases with age and is significantly reduced in the 2 yr old WT versus 2 mo old WT. Note that D3KO fractional shortening is significantly reduced from age-matched WT but similar to that of the 2 yr old WT. **C.** Stroke volume peaks in WT at 1 yr before returning at 2 yrs to similar values as in 2 mo old WT. In D3KO stroke volume is significantly decreased over age-matched WT controls. **D.** Similar to stroke volume, cardiac output in WT significantly increases from 2 mo to 1 year before declining at 2 yrs of age. In D3KO cardiac output is significantly reduced compared to 1 yr old WT (p<0.001, ANOVA), or when solely compared to age-matched WT controls (p = 0.04, t-test).

To determine if aging-related changes in blood pressure and heart rate were related to alterations in cardiac function, we next performed ultrasound echocardiograms in anesthetized animals. Cardiac echograms were analyzed from 2 mo (n = 9), 1 yr (n = 7), and 2 yr old WTs (n = 6), and 2 mo old D3KOs (n = 5), and data are reported in Figures 2 and 3. Overall, six morphological (Figure 2) and four functional parameters (Figure 3) were measured or calculated and compared between all groups.

Systolic diameter (Fig. 2A1) gradually increased with age in WT from 2.2±0.2 mm at 2 mo to 2.9±0.2 mm at 2 yr (p = 0.029), however we did not find any difference between 2 mo old D3KO (2.36±0.06 mm) and age-matched WT. Measurements for systolic volume (Fig. 2B1) displayed a similar trend: from 18.6±3.5 µl in 2 mo WT to 32.4±3.1 µl in 2 yr old WT (p = 0.024), while systolic volume in the 2 mo old D3KO (20.2±0.9 µl) remained similar to the age-matched WT.

Diastolic diameters (Fig. 2A2) only slightly and insignificantly increased with age in WT, from 3.5±0.17 mm at 2 mo to over 4.05±0.21 mm at 1 yr to 3.95±0.2 mm at 2 yr. Diastolic diameters of 2 mo D3KO were again similar to their age-matched WT controls (3.3 mm±0.1 mm), which were significantly smaller than the 1 yr WT (p = 0.037, Fig. 2A2). Similar to the trends observed between systolic diameter and volume, diastolic volume displayed a trend towards an increase in aging WT (2 mo: 44.28±6.2 µl; 1 yr: 89.6 µl±8.5 µl; 2 yr: 58.8±8.8 µl), but was similar between age-matched WT and D3KO (D3KO: 44.5±3.53 µl). However, the difference between 1 yr old WT and 2 mo D3KO was significant (p = 0.04, Fig. 2B2).

With age, LV mass increased in WT, from 94.7±4.6 mg at 2 mo to 132.1±7.9 mg at 2 yr (Fig. 2C1). LV mass was similar, if slightly reduced, in 2 mo D3KO compared to age-matched WT controls (74.5±4.4 mg), and was significantly lower than those of 1- and 2 yr WT (p = 0.001).

To account for the increase in body weight of the WT animals with age, we adjusted LV mass to body weight (Fig. 2C2). We found that in WT the LV mass/body weight ratio remained stable with age (2 mo: 0.35±0.02%; 1 yr: 0.33±0.03%; 2 yr: 0.38±0.02%), but was significant decreased in the young D3KO compared to age-matched and 2 yr old WTs (p = 0.001). These findings suggest that the hypertension observed in young D3KO is not associated with anatomical changes in the hearts of these animals or with cardiac muscle hypertrophy. We therefore next assessed the functional parameters of cardiac function in WT and D3KO.

In WT mice, ejection fraction (Figure 3A) significantly decreased with age from 2 mo (68±3.2%) over 1 yr (61.7±1.6%) to 2 yrs (52.8±3.9%; p = 0.01). In D3KO, ejection fraction (54±4.3%) was significantly smaller than in age-matched WT (p = 0.01) but similar to that of the 2 yr WT cohort. Fractional shortening (Figure 3B) displayed a similar trend, with values significantly decreasing with age in WTs from 37.8±2.5% at 2 mo to 27.1±2.5% at 2 yr (p = 0.012). Again, values for 2 mo D3KOs mimicked those of old WT and were significantly decreased from age-matched WTs (28.2±2.9%, p = 0.012).

**Figure 4:**
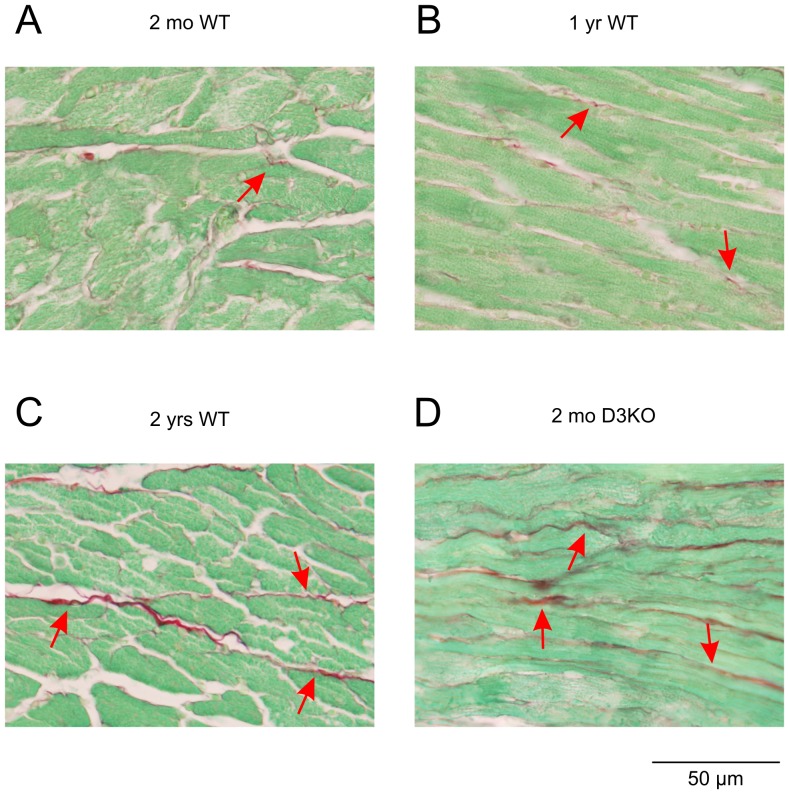
Myocyte interstitial fibrosis with age. Representative images of the left ventricle myocardium of 2 mo old WT (**A**), 1 yr old WT (**B**), 2 yr old WT (**C**), and 2 mo old D3KO mice (**D**). Images show increased content and thickness of fibrillar collagen with age (arrows) in WT hearts and in young D3KO hearts.

Unlike ejection fraction and fractional shortening, stroke volume did not change significantly with age in WT mice (Figure 3C). We observed a slight increase from 2 mo to 1 yr, but no significant differences between young and old WT (2 mo: 34.4±2.7 µl; 1 yr: 45±4.7 µl; 2 yr: 37.4±7.1 µl). In contrast, stroke volume in the young D3KO (24.7±3.7 µl) showed a strong trend (p = 0.054) for a decrease over age-matched WT. Assessing cardiac output (Figure 3D), we observed in WT a significant increase from 2 mo (12.5±0.9 ml/min) to 1 yr (21.1±2.1 ml/in, p<0.001), followed by decrease at 2 yr (15.3±2.8 ml/min). Cardiac output of D3KO (8.8±1.4 ml/min) was significantly lower than that of the 2 mo WT (p = 0.04, t-test). These data suggest that cardiac dynamic function appears to be compromised in young D3KO compared to age-matched WT controls. The numerical data from figures 3 and 4 are summarized in Table 2.

**Table 2:** Echocardiogram analysis.

	2 mo WT	1 yr WT	2 yrs WT	2 mo D3KO
Systolic diameter (mm)	2.22±0.56	2.73±0.45 ♯	2.83±0.26 §	2.21±0.25 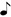
Diastolic diameter (mm)	3.51±0.52	4.05±0.57	3.9±0.38	3.26±0.25 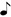
Ejection fraction (%)	68±3.2	61.65±1.6	53.18±3.9 §	55±2.9 €
Fractional shortening (%)	37.76±2.5	32.84±1	27.39±2.6 §	28±2.9 €
LV mass (mg)	94.66±13.88	138.52±32.56 ♯	133.64±17.38 §	72.29±10.67 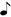 , 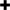
LV mass/body weight (%)	0.35±0.05	0.33±0.08	0.38±0.05	0.25±0.04 €, 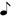 , 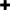
Systolic volume (µl)	18.6±10.57	29.21±10.71 ♯	31.11±6.57 §	18.05±3.05 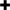
Diastolic volume (µl)	52.99±18.5	74.28±22.62 ♯	67.17±15.85	43.81±8.31 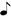 , 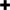
Stroke volume (µl)	34.39±8.2	45.07±12.41	36.06±14.31	25.76±7.96 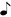
Cardiac output (ml/min)	12.55±2.8	21.14±5.52 ♯	14.59±5.23	9.32±3.14 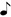 , 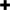

Average values ±S.E.

♯  =  significant difference between 2 mo WT and 1 yr WT.

§ =  significant difference between 2 mo WT and 2 yrs WT.

€  =  significant difference between 2 mo WT and 2 mo D3KO.

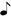
  =  significant difference between 1 yr WT and 2 mo D3KO.

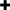
  =  significant difference between 2 yr WT and 2 mo D3KO.

### Morphometric analyses

We next assessed the morphometric features of the WT and D3KO hearts and measured LV total area and chamber area and MCSAs of both the endo- and epicardium (see Table 3). We found aging-related increases in WT for LV total area and LV chamber area, but no significant differences between age-matched WT and D3KO. Both LV total area and chamber areas were significantly smaller in D3KO than in 2 yr WT (p = 0.009 and p = 0.007, respectively. Similarly, in WT, MCSA of both endo- and epicardial areas increased significantly from 2 mo to 1 yr of age, and the 1 yr WT animals were significantly different from 2 mo D3KO (p<0.001), but we did not detect any differences between age-matched WT and D3KO. These data suggest that the increase in blood pressure and the reduction in heart rate and cardiac function observed in D3KO are not caused by changes in MCSA and LV size.

**Table 3:** Morphometric analysis.

	2 mo WT	1 yr WT	2 yrs WT	2 mo D3KO	*ANOVA*
LV area (mm^2^)	15.79±1.62	20.38±0.87	20.2±1.3	15.02±0.76 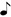 , 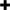	P = 0.009
LV chamber area (mm^2^)	2.11± 0.05	2.24±0.22	2.58±0.21	1.51±0.09 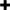	p = 0.007
MCSA epicardium (mm^2^)	230.2±13.58	323.96 ±16.54 ♯	267.8 ±12.67	230.93 ±13.52 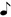	p<0.001
MCSA endocardium (mm^2^)	284.3±13.98	340.93±12.57 ♯	254.72±9.66	265.37±8.26 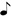	p<0.001

Average values ±S.E.

♯  =  significant difference between 2 mo WT and 1 yr WT.

¶ =  significant difference between 1 yr WT and 2 yrs WT.

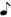
  =  significant difference between 1 yr WT and 2 mo D3KO.

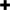
  =  significant difference between 2 yr WT and 2 mo D3KO.

### Histological analyses

**Figure 5:**
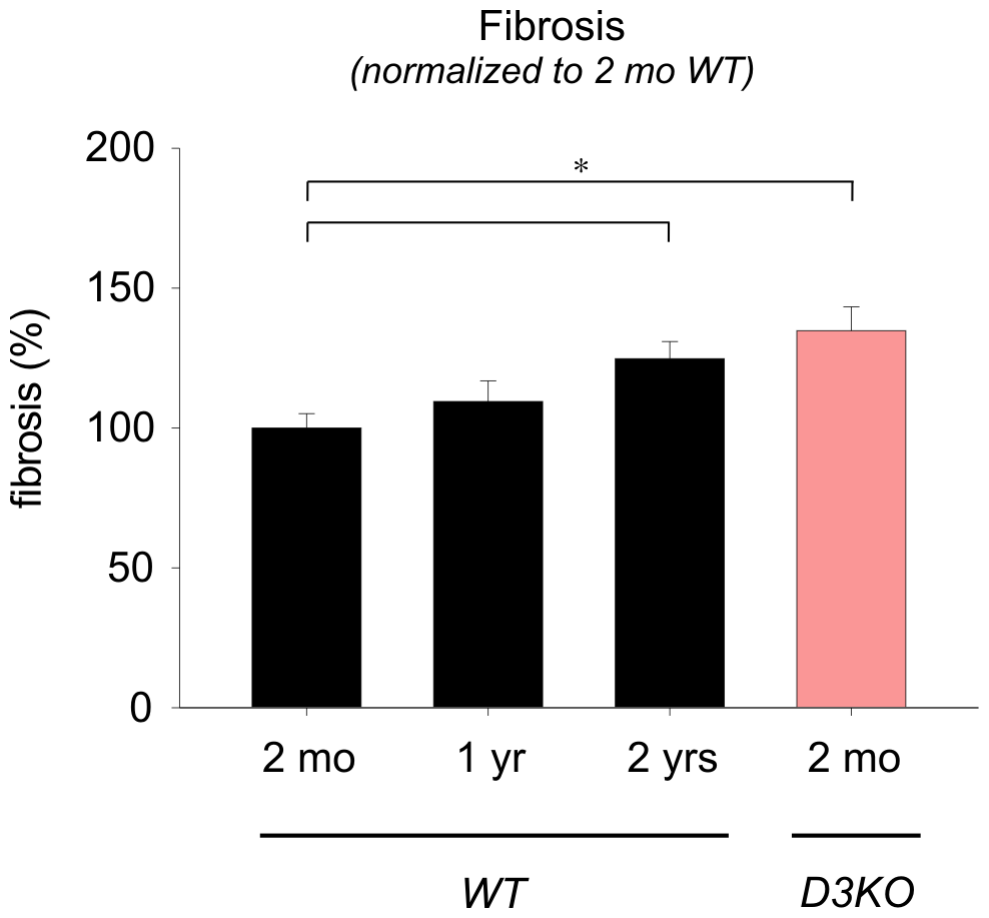
Quantification of myocyte interstitial fibrosis with age. Levels of fibrosis gradually rise with age in the WT hearts and are significantly increased in the 2 yr old WT hearts compared to the 2 mo old WT. Fibrosis is also significantly increased in 2 mo old D3KO hearts over age-matched WT and similar to levels observed in the 2 yr old WT.

As cardiac fibrosis is a hallmark of heart disease and hypertension [7], [34], we next assessed fibrosis and fibrotic collagen in the heart (Figure 4). In 2 mo WTs, we detected low levels of interstitial fibrosis (Figure 4A, arrow), and with increasing age, fibrosis-positive labeling strongly increased (Figure 4B, C, arrows). Surprisingly, in 2 mo D3KO hearts, fibrosis labeling was stronger than in age-matched WTs and was comparable to the labeling observed in the 2 yr WT (Figure 4D, arrows). Quantification of the histological data (Figure 5) revealed significant increases in total myocardial fibrosis with age in the WT animals (by, at 1 yr: 9.5±7.3%; 2 yr: 24.8±6.1% versus 2 mo WT; p = 0.002). Importantly, cardiac fibrosis expression in 2 mo D3KO was also significantly increased over age-matched WTs (by 34.8±8.5%, p = 0.002, Figure 5). These data suggest that increased interstitial fibrosis may play a critical role in the emergence of hypertension and bradycardia in the young D3KOs.

### Western blot and zymographic analyses

**Figure 6:**
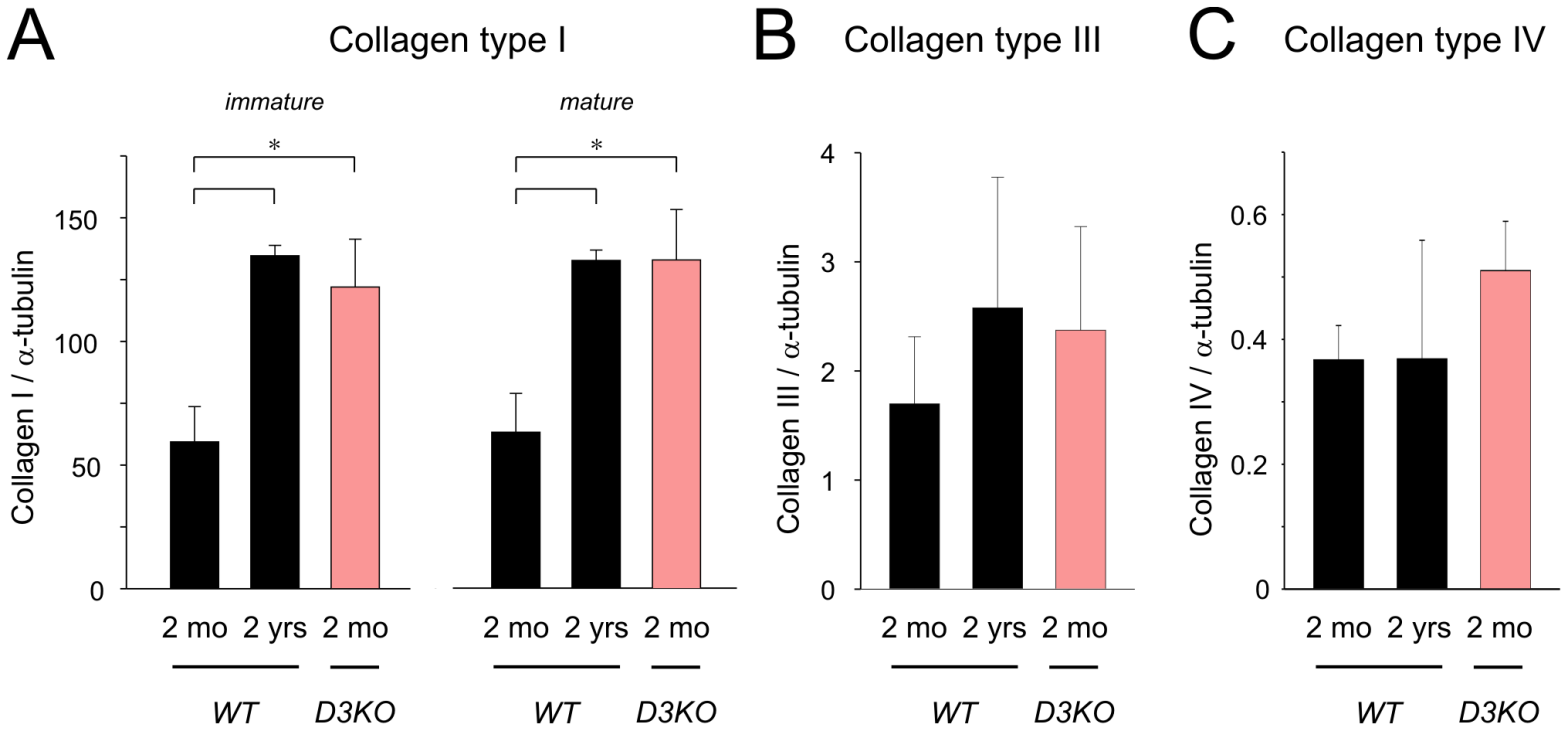
Quantification of Western blot analyses for collagen types-I, type-III, and type-IV, normalized to α-tubulin. **A.** Collagen-type I: expression levels of immature (pro) and mature forms of collagen type-I increase significantly with age in WT hearts and are similar between 2 yr old WT and 2 mo old D3KO. **B.** Collagen type-III: expression levels of collagen type III were similar in young and old WT hearts and were not significantly different in D3KO. **C.** Collagen type-IV: as with collagen type-III, expression levels of collagen type-IV were similar in the hearts of young and old WT and D3KO.

Interstitial fibrosis partly arises from an increased deposition of collagen in the extracellular space [7], and of the six collagen types found in the heart, collagen type-I, type-III, and type-IV are most commonly associated with fibrosis [34], [35], [36]. As a wide range of mechanisms have been associated with cardiac fibrosis (e.g. reactive oxygen species, angiotensin or endothelin signaling pathways, growth factor activation), we focused here on the link between collagen and matrix-metalloproteases (MMPs), using immunoprecipitation and Western blotting approaches. We found significant increases of both pro- and mature collagen type I with age in WT hearts (p = 0.002 and p = 0.005, respectively, Figure 6A). Furthermore, levels of both pro- and mature collagen type-I in the 2 mo D3KO were similar to 2 yr WT and significantly increased over age-matched WT (p = 0.002, Figure 6A). In comparison, expression of collagen type-III (Figure 6B) or type-IV (Figure 6C) did not change significantly with age in WT hearts, nor were they significantly modified in the 2 mo D3KO compared to their age-matched WT controls.

**Figure 7:**
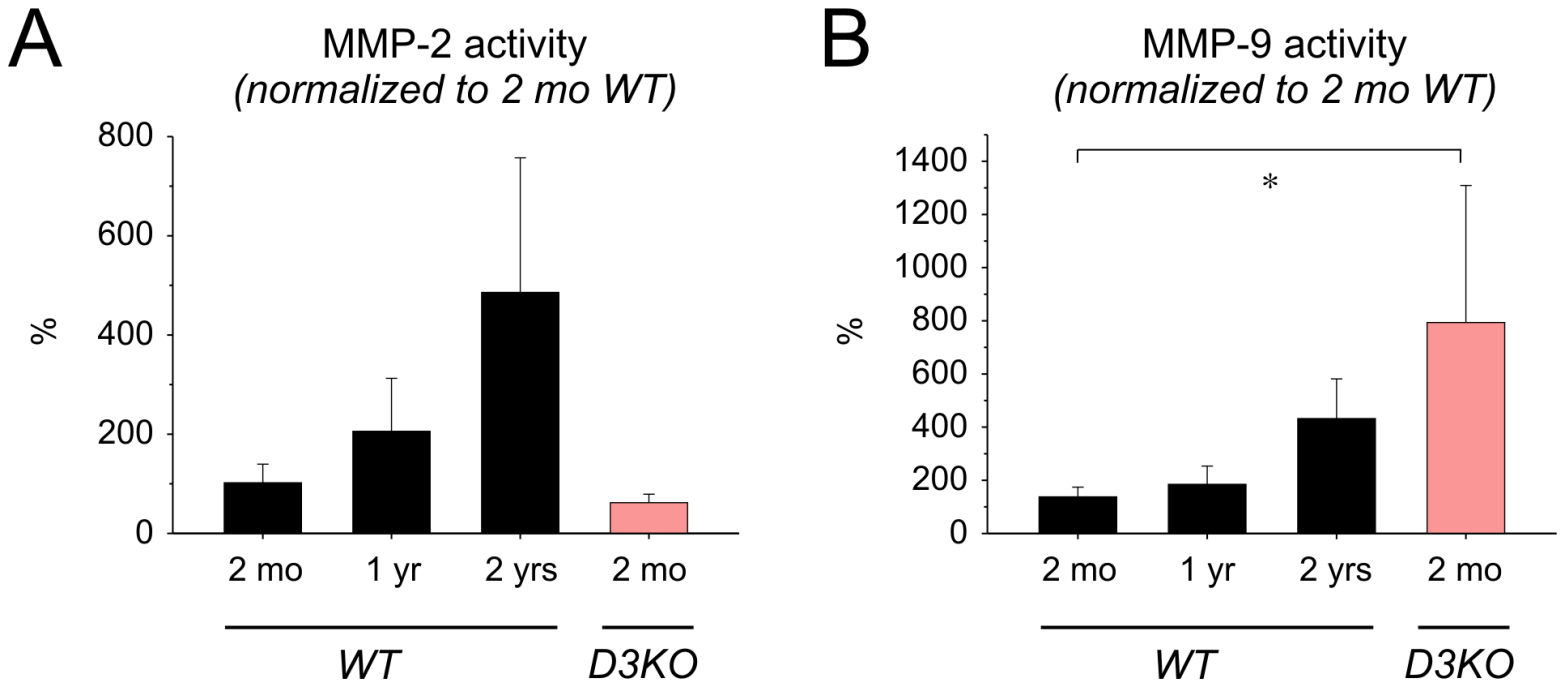
Quantification of matrix metalloproteinase activities. **A.** MMP-2: In WT, MMP-2 activity increased with age and was similar between age-matched WT and D3KO. **B.** MMP-9: as with MMP-2, MMP-9 activity increased with age in WT, but was significantly higher in young D3KO compared to WT hearts at any age (ANOVA).

To test whether the increased expression of collagen type I was affiliated with parallel changes in matrix metalloproteinase (MMP) activity, we assessed myocardial activity of MMP-2 [37] and MMP-9 [38] (Figure 7). In WT hearts, MMP-2 activity showed a strong trend to increase with age, but activity levels of 2 mo WT and 2 mo D3KO were comparable. Similarly, MMP-9 activity increased with age in WT, but MMP-9 levels were significantly higher in D3KO compared to all WT cohorts (p = 0.022, Figure 7B). Together, these data suggest that the aging-related changes in hypertension and bradycardia observed in old WT are already present in young D3KO, and they indicate that these changes with age and in D3KO may be related to an increased interstitial fibrosis in the heart, possibly mediated by collagen type-I and MMP-9 pathways.

### Animal survival

**Figure 8:**
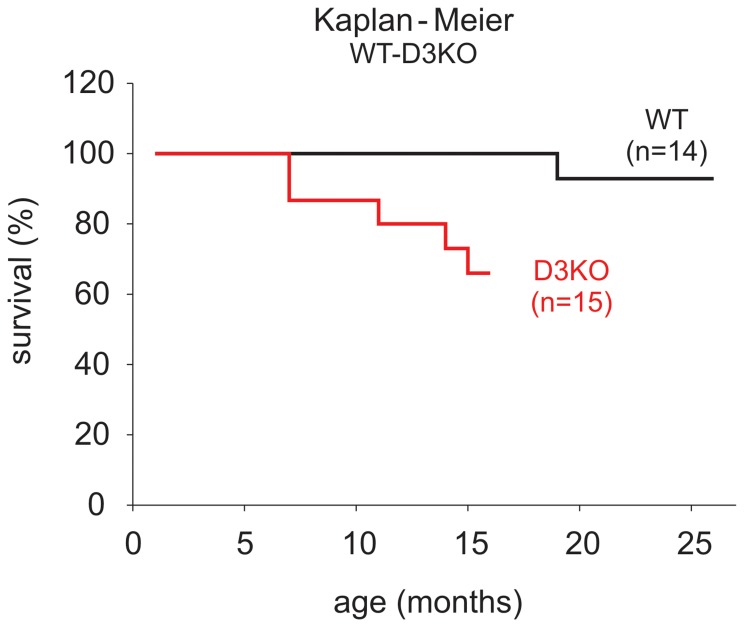
Kaplan-Meier curve of survival for WT and D3KO animals. Two cohorts of male animals kept were maintained and expressed a differential survival profile. D3KO started to die prematurely at 7 months of age, and at 15 months of age, only 67% (10/15) D3KO mice survived while 100% (14/14) of the WT animals survived to this time point.

As our previous data suggested that a dysfunction of the D3 receptor is associated in young D3KO with an increase in blood pressure and cardiac fibrosis observed otherwise only in aged WT animals, we wanted to assess the physiological importance of D3 receptor function on life span. As shown in Figure 8, a Kaplan-Meier survival analysis indicates a reduced life span for the D3KO animals. Specifically, D3KO mice started prematurely dying at about 7 months of age, either due to terminal morbidity or mortality, and at 16 months of age only 67% D3KO mice had survived (10 out of 15 animals). In contrast, all 14 WT were still alive by this time. These data strongly suggest that a systemwide dysfunction of the D3 receptor not only affects cardiac function, but also severely shortens life span.

## Discussion

We present here compelling evidence that a dysfunction of the dopamine D3 receptor is associated with hypertension and myocardial interstitial fibrosis and that these consequences mimic aging-related alterations in cardiac remodeling and function, possibly impacting life span.

Using non-invasive blood pressure measurements and functional echocardiography, together with morphometric analyses, histology, protein expression and activity assays *in vitro*, we found that aging-related changes of cardiac function and remodeling observed in aged (2 yr old) WT are largely mimicked in young (2 mo old) D3KO animals.

Specifically, systolic and diastolic blood pressures and heart rates in 2 mo old D3KO were significantly increased over age-matched WT controls but similar to those of the 2 yr old WT group. Echocardiography failed to reveal morphological differences between age-matched young WT and D3KO, but did demonstrate a decline of function in fractional shortening and ejection fraction in young D3KO that mimicked those of old WT animals. Subsequent histological analyses revealed an aging-related increase in interstitial fibrosis in the myocardium of WT, with fibrosis in the 2 yr WT equivalent to that observed in the 2 mo D3KO. Western blot analyses suggest that this aging-related fibrosis in WT might be due to increased expression of collagen type I, which was also significantly elevated in the D3KO over its age-matched control. Lastly, activities of MMP-2 and MMP-9 increased with age in WT; however, only MMP-9 activity both increased in WT with age and was similar in D3KO to that in 2 yr old WT. Together, these comprehensive findings attest that the dopamine D3 receptor is critical in maintaining cardiac health and homeostasis, and in turn that the dysfunction of the D3 receptor results in exaggeration of an aging phenotype in the heart that appears to be related to the collagen type-I/MMP-9 signaling cascade.

An elevation in blood pressure in aging humans has been well documented; however, the underlying causes for the development of hypertension with age remain uncertain. Proposed mechanisms center around pathologic renal defects, such as alterations in the renin/angiotensin system [39], [40], SNS dysfunction [41], or abnormalities during cardiovascular development. All of these etiologies potentially contribute to the development of hypertension with age [42].

During the early pathogenesis of hypertension, an increase in cardiac output subsequently leads to increased vascular resistance, and these events appear to be primarily neurogenic in origin [41], [43], [44]. In contrast, in long-term hypertensive patients, blood volume and cardiac output generally return to normal levels, but vascular resistance remains elevated possibly through structural, non-neurogenic mechanisms. This division between the stages of hypertension is important in determining the underlying mechanisms, and our findings point to a role for the D3 receptor in the emergence of hypertension in its chronic form.

Dopamine is a potent neurotransmitter that also markedly contributes to the control of cardiac and vascular function, including heart rate variability (HRV), which is considered a measure of autonomic regulation of cardiac activity [45], [46]. We recently reported that treatment with a D3 receptor agonist improved HRV in patients with Restless Legs Syndrome (RLS) [47], suggesting that activation of this inhibitory dopamine receptor can boost both neurological and autonomic functions. While our tail-cuff method to measure blood pressure and heart rate did not provide a sufficiently-fast time resolution to directly measure HRV, and while we abstained in this study from interventional approaches to directly measure HRV or cardiac pressures and volumes, our survival data suggest that a dysfunction of the D3 receptor is affiliated with a risk for a decreased life span. The D3KO mouse, originally characterized phenotypically as hyperactive with elevated locomotor activity, reduced anxiety, and hypertension [16], [48], has been previously proposed as a model for RLS [49]. Interestingly, patients suffering from RLS often present clinically with high blood pressure and cardiovascular disease [50], [51]. Moreover, the severity of RLS pathology and symptomatology worsen with age, and treatment with D3-receptor agonists can remediate RLS symptoms including hypertension [52], [53]. These findings establish a basis for our theory that the D3 receptor system plays a pivotal role in the emergence of aging-related hypertension and suggest that the D3KO mouse may also serve as a new animal model with which to study the mechanisms affiliated with aging-related hypertension and cardiac remodeling.

In many tissues, a structural reorganization and enhancement of matrix proteins including collagen, fibronectin, and proteoglycans occur with age [54], [55]. Such fibrotic changes often result in rigidity and stiffening of the tissue with loss of functional integrity. We found that myocardial interstitial fibrosis increased (∼30%) with age in WT animals, corresponding to expected age-related fibrotic modifications. In comparison, in young D3KO mice myocardial interstitial fibrosis was ∼40% higher than that of age-matched WT controls and comparable to the 2 yr old WT. In support of the fibrosis data, IP-Western analyses of myocardial homogenates revealed that pro- and mature collagen type-I but not type-III or type-IV significantly increased with age in the WT animals. In a similar fashion, young D3KO expressed significantly elevated levels of pro- and mature collagen type-I, comparable to that of the 2 yr old WT. A decline of the heart function with aging and age-related cardiac fibrotic remodeling has been well characterized [7], [34]. Myocardial collagen accumulation has also been identified as a major contributor to these aging-related changes. Here, we identify collagen type-I as the major component in aging-related myocyte fibrosis in WT animals, supporting previous observations that collagen type-I is a primary collagen isotype in the aged heart [35]. Collagen type-I expression was significantly increased in the young D3KO and similar to levels found in 2 yr old WT. Interestingly, an inverse relationship between fractional shortening and myocardial collagen content has been recently suggested to contribute to aging-related fibrotic remodeling [56]. Additionally, although several studies have examined the role for the D1 and/or D2 receptors on collagen deposition following coronary artery occlusion [57] or under conditions of experimental arthritis [58], this is the first report highlighting a role for the D3 receptor in myocardial collagen accumulation.

It is possible that, in addition to these measures of cardiac remodeling, neural remodeling including changes in cardiac neuron size, density, branching and/or fibrosis could potentially contribute to the early onset of cardiac dysfunction observed in the D3KO mice, and possibly also play a role in the decreased life span of these animals. A monoamine receptor-mediated remodeling of cardiac neurons has been recently observed in chronic heart disease [59], and it is conceivable that the persistent dysfunction of the D3 receptor could also entail similar consequences. Another intriguing hypothesis is that the increase in cardiac fibrosis could also affect the cytoskeleton of cardiac myocytes themselves, and that these pathological stimuli might corroborate or induce maladaptive remodeling of cardiac neurons [60]. Future experiments will be designed with these hypotheses in mind, to dissociate the possible influence of altered cardiac neurons and fibrosis in the emergence of the D3KO cardiac and autonomic phenotype.

Considering the regulatory roles for matrix-degrading MMPs in fibrosis, we found that the activities of the gelatinolytic MMP-2 and MMP-9 increased with age in WT cardiomyocyte homogenates. Interestingly, D3KO showed comparable levels of MMP-2 activity to those of age-matched WT, yet their MMP-9 activities were significantly elevated over all WT cohorts, nearly 8-fold higher than age-matched WT and doubling levels observed in the 2 yr old WT. Although a correlation between modulation of D2 or D5 receptors and MMP-9 synthesis has been previously suggested [61], [62], to date no study has reported an association between D3 receptor deficiency and MMP-9. An increase of MMP-9 levels has been recently suggested as a potential plasma marker for cardiac aging [63], and deletion of MMP-9 in MMP-9 null mice attenuated the age-related decline in diastolic function [64]. Thus the enhanced MMP-9 activity and the elevated collagen type-I expression observed in the hearts of young D3KO suggest that both are involved in the early establishment of the fibrotic phenotype also present in old WT.

It is intriguing to note that young D3KO display a lack of cardiac ventricular hypertrophy and remodeling despite developing increased levels of systolic and diastolic blood pressure. The conventional notion is that hypertension stimulates compensatory cardiac remodeling and therefore is usually associated with cardiac hypertrophy [65], [66]. However, the lack of overt differences in LV dimensions and MCSAs between D3KO and their age-matched WT argues that the D3KO mouse may represent a unique model in which hypertension and interstitial fibrosis can be separated from cardiac hypertrophy.

## Conclusions

Taken together, our data provide sound evidence that dysfunction of the dopamine D3 receptor is associated with emergence of aging-related hypertension and changes in cardiac function and life span. These data are consistent with the theory that the D3KO mouse heralds a new animal model with which to understand the role of the dopamine D3 receptor in aging heart function, and in particular its role in cardiac remodeling associated with hypertension.
